# Confidence, Acceptance and Willingness to Pay for the COVID-19 Vaccine among Migrants in Shanghai, China: A Cross-Sectional Study

**DOI:** 10.3390/vaccines9050443

**Published:** 2021-05-02

**Authors:** Kaiyi Han, Mark R. Francis, Ruiyun Zhang, Qian Wang, Aichen Xia, Linyao Lu, Bingyi Yang, Zhiyuan Hou

**Affiliations:** 1 School of Public Health, Fudan University, Shanghai 200032, China; kaiyi.han@lshtm.ac.uk (K.H.); 17301020124@fudan.edu.cn (R.Z.); 19211020072@fudan.edu.cn (Q.W.); 16301050086@fudan.edu.cn (A.X.); 17301020062@fudan.edu.cn (L.L.); 2 Department of Infectious Disease Epidemiology, London School of Hygiene & Tropical Medicine, London WC1E 7HT, UK; 3 Health Sciences Unit, Faculty of Social Sciences, Tampere University, 33014 Tampere, Finland; mark.francis@tuni.fi; 4 Division of Epidemiology and Biostatistics, The University of Hong Kong, Hong Kong 999077, China; yangby@hku.hk; 5 NHC Key Laboratory of Health Technology Assessment, Fudan University, Shanghai 200032, China

**Keywords:** COVID-19, vaccine, acceptance, intention, confidence, China

## Abstract

Understanding the public’s attitude towards COVID-19 vaccination and their acceptance could help facilitate the COVID-19 rollout. This study aimed to assess the acceptance and willingness to pay (WTP) for the COVID-19 vaccine among migrants in Shanghai, China. A cross-sectional study was conducted among 2126 migrants in Shanghai for the period 1–20 November 2020. Convenience sampling was used to recruit respondents in workplaces with large numbers of migrant workers. Multivariable (ordered) logistic regressions were used to examine factors associated with acceptance and WTP of the COVID-19 vaccine. Most (89.1%) migrants would accept COVID-19 vaccination. Over 90.0% perceived the COVID-19 vaccine as important, while only 75.0% and 77.7% perceived vaccines safe and effective. Socio-demographic factors were not significantly associated with vaccine acceptance, but confidence in the importance (OR 8.71, 95% CI 5.89–12.89), safety (OR 1.80, 95% CI 1.24–2.61) and effectiveness (OR 2.66, 95% CI 1.83–3.87) of COVID-19 vaccine was significantly positively associated with vaccine acceptance. The top reasons for vaccine hesitancy were lack of vaccine information and confidence. The proportion of those definitely willing to get the COVID-19 vaccine was 20% lower if paid by themselves than free vaccination. Migrants were willing to pay a median amount of USD 46 for the COVID-19 vaccine. Results show that a high acceptance of the COVID-19 vaccine was universal among migrants in Shanghai. Concerns about vaccine safety, effectiveness and high costs of the COVID-19 vaccine may hinder their uptake. Effective health communication to build confidence in the COVID-19 vaccine and subsidies toward the costs of these vaccines are needed to improve uptake.

## 1. Introduction

The coronavirus disease 2019 (COVID-19) has caused around 140 million cases and 3 million death worldwide as of April 2021 [1]. Since no effective antiviral treatment for COVID-19 is currently available, vaccination against COVID-19 is essential to controlling the pandemic [2]. The COVID-19 pandemic has triggered intense global research and development activities aimed at developing effective vaccines against the disease. As a result, 88 vaccine candidates with multiple techniques have entered into clinical trials as of April 2021 [3]. Several vaccines have finished their phase III clinical trials, and have been approved for use in many countries [4]. In China, the government authorized the emergency use of the COVID-19 vaccine in October 2020, which is voluntary and costs approximately Chinese Yuan (CNY) 400 (USD 61), and further approved for use on 30 December 2020 [5].

The success of vaccination programs relies on high vaccination uptake [6]. However, recent outbreaks of vaccine-preventable diseases such as measles [7,8], and poliomyelitis [9], point toward pockets of under-vaccinated or non-vaccinated populations in different regions around the world [10]. The under-vaccinated are partly associated with personal strong anti-vaccination convictions [11], while a larger proportion is potentially hesitant towards vaccination [12]. In 2019, vaccine hesitancy was listed by the World Health Organization as one of the top ten threats to global health [13]. Growing concerns among the public regarding the safety and effectiveness of vaccines are considered a significant factor influencing individual vaccination behavior [14]. Therefore, monitoring public confidence and acceptance towards the recently introduced COVID-19 vaccines could aid to identify the barriers for vaccine uptake and guide the communication interventions to ensure high vaccination uptake during the pandemic.

Several studies have investigated public confidence and acceptance of COVID-19 vaccines and reported substantial heterogeneity across countries [15]. Around 58% to 69% of surveyed adults were willing to get a COVID-19 vaccination in the United States [16,17,18,19], while the proportion was reported as 62% in France and 80% in Denmark and the United Kingdom [20]. A slightly higher acceptance was reported in China in March 2020, with over 90% of adults accepting a COVID-19 vaccination [21].

However, most previous surveys focus on general populations, often excluding vulnerable groups such as migrants and other low socioeconomic groups. In China, the population of internal migrants (people who leave their birthplaces to seek jobs in cities) increased dramatically as a consequence of rapid urbanization since the 1980s, and reached approximately 286.5 million in 2017 [22]. Previous studies suggest that the health of migrants is worse than non-migrants in urban areas because of limited social welfare, low socioeconomic status, risk of discrimination, and marginalization [23,24,25]. In addition, most migrants are engaged in the service industry [26], and it is likely that their risk of COVID-19 infection may be higher due to close and frequent contact with the public. In the present study, we aimed to assess migrants’ acceptance and willingness to pay for the COVID-19 vaccine, and their determinants. This research can help inform strategies to facilitate COVID-19 vaccine rollout among vulnerable populations.

## 2. Materials and Methods

### 2.1. Study Design

We conducted a cross-sectional, web-based survey among a sample of internal migrant workers in Shanghai during 1–20 November 2020. In Shanghai, internal migrants (9.78 million) account for 40.27% of the total population, and the service industry where most migrants work accounts for 72.74% of its gross domestic product (GDP) [27]. We identified 23 workplaces with large numbers of migrant workers as our study sites in different communities in Pudong, Minhang and Xuhui districts, including food market or supermarket, services industry such as hotel, catering or express delivery, and manufacturing industry such as factory. In selected workplaces, all migrants were invited to complete a questionnaire after confirming whether their residence registration is local or not, and we also encouraged respondents who completed the survey to disseminate the survey link to all their contacts.

### 2.2. Data Collection

A web-based questionnaire was developed using the Questionnaire Star [28], a paid website that helps generate, distribute and retrieve electronic questionnaires on the mobile platform. Respondents could access the questionnaire through WeChat, a social media with 1.1 billion active users [29]. Each WeChat account was allowed to fill in the questionnaire once to avoid data duplication. Respondents could also share the link of the questionnaire via social media platforms to invite their colleagues or friends to participate. The questionnaire was pilot tested among 10 respondents in a non-study community. It took approximately 5 min to complete the self-administered questionnaire, and respondents received electronic currency worth Chinese Yuan (CNY) 5 (USD 0.7) as a gift after they completed the questionnaire.

In total, 3771 migrants accepted our invitation to participate in the survey. Among these respondents, 1174 questionnaires were not completed, and 471 questionnaires were completed in less than 100 s (which was the minimum time considered valid to complete our questionnaire in the pilot survey) or had missing data, which were excluded from the analyses. A total of 2126 respondents with valid data were included for analysis in our study.

### 2.3. Instruments

Acceptance of COVID-19 vaccine was measured with the question, “When a COVID-19 vaccine becomes available, will you get vaccinated?” and this question was asked three times: without conditions, conditioned if it is recommended and free, and conditioned if it is recommended and paid out of pocket [19,21,30]. Response options were “definitely yes”, “possibly yes”, “not sure”, “possibly no”, and “definitely no”. Respondents who responded “yes” (accept to be vaccinated) were further asked one question “when do you hope to get vaccinated?” and respondents who responded “no” or “not sure” (hesitant to be vaccinated) were asked to provide reasons for vaccine hesitancy. We also assessed respondents’ willingness to pay (WTP) for the COVID-19 vaccine with the question, “what is the maximum amount you are willing to pay for COVID-19 vaccine” [19,21,30].

Perceived susceptibility to vaccine-preventable diseases and vaccine confidence have been considered as key determinants for vaccine acceptance [31]. Confidence towards the COVID-19 vaccine is measured by the following three statements from Vaccine Confidence Index: “COVID-19 vaccines are important for people to have”, “overall I think COVID-19 vaccines are safe”, and “overall I think COVID-19 vaccines are effective” [32]. Each respondent was asked to rate the extent to which they agreed with these statements on a five-point Likert scale: strongly agree, tend to agree, not sure, tend to disagree, strongly disagree. We also assessed the probability of perceived susceptibility to COVID-19 on a five-point Likert scale: very high, high, not sure, low, very low.

### 2.4. Statistical Analysis

Descriptive statistics were performed for general characteristics of respondents, and their confidence and acceptance of the COVID-19 vaccine. Taking the maximum amount respondents were willing to pay for COVID-19 vaccine as their WTP, we calculated the proportion of respondents who were willing to accept vaccination at various price points (CNY 0, CNY 50, CNY 100, and then CNY 100 increments to a maximum of CNY 1000) of WTP, and cumulative proportion of respondents with willingness at each price was also calculated to investigate their demand for COVID-19 vaccine. Respondents’ characteristics were compared between those accepting and hesitant to be vaccinated against COVID-19 using Pearson’s Chi-square test. We performed logistic regression to examine the factors associated with acceptance of the COVID-19 vaccine, and ordered logistic regression to examine factors associated with WTP for the COVID-19 vaccine. In basic regression models, only demographic and socio-economic factors (gender, age, marital status, number of family members, education, income, years of local residence, workplace, frequency of contact with local residents, and health status) were included as independent variables; in additional models, perception of susceptibility to COVID-19 and vaccine confidence (in the importance, safety and effectiveness of COVID-19 vaccines) was further added. Odds ratios (ORs) and 95% confidence intervals (CIs) were reported. All analyses were performed with STATA, version 14.0 (Stata Corp, College Station, TX, USA).

## 3. Results

### 3.1. Sample Characteristics

Among the 2126 respondents with valid data, around half were female, aged 26–35 years old, and lived in families with 2–3 members (Table 1). Around 70% of internal migrants had married and lived in Shanghai for at least one year. Nearly half of migrants obtained college or bachelor level education, and 71% had a monthly income of less than CNY 7500 (USD 1160). They worked at food markets or supermarkets, the small service industry, the manufacturing industry, companies or government agencies, or other places. Around 54% of migrants reported having frequent contact with the local residents. The majority (80%) considered their health status to be good.

### 3.2. Acceptance, Willingness to Pay and Confidence of COVID-19 Vaccine

**Figure 1:**
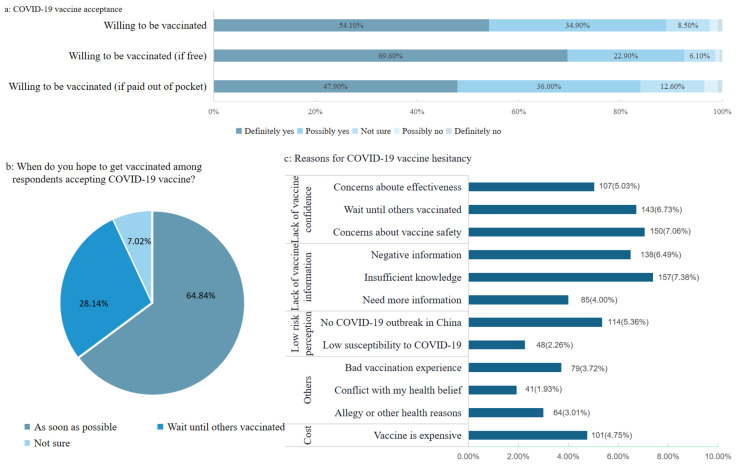
Acceptance of COVID-19 vaccine: (**a**) the proportions of migrants who are willing to be vaccinated against COVID-19; (**b**) When migrants hope to get vaccinated for respondents who are willing to be vaccinated against COVID-19; (**c**) Reasons for COVID-19 vaccine hesitancy for respondents who are unwilling or unsure to be vaccinated against COVID-19.

Most respondents (*n* = 1894, 89.09%) reported that they would definitely or possibly accept a COVID-19 vaccination if one is successfully developed and approved for listing (Figure 1a). Among the respondents who reported that they would accept vaccination against COVID-19, 64.84% hoped to get vaccinated in a timely manner, and 28.14% would wait until others have been vaccinated (Figure 1b). The top reasons cited by the respondents who were unsure or not willing to be vaccinated against COVID-19 (*n* = 232), included concerns about vaccine safety or effectiveness, and lack of information about the COVID-19 vaccine (Figure 1c).

**Figure 2:**
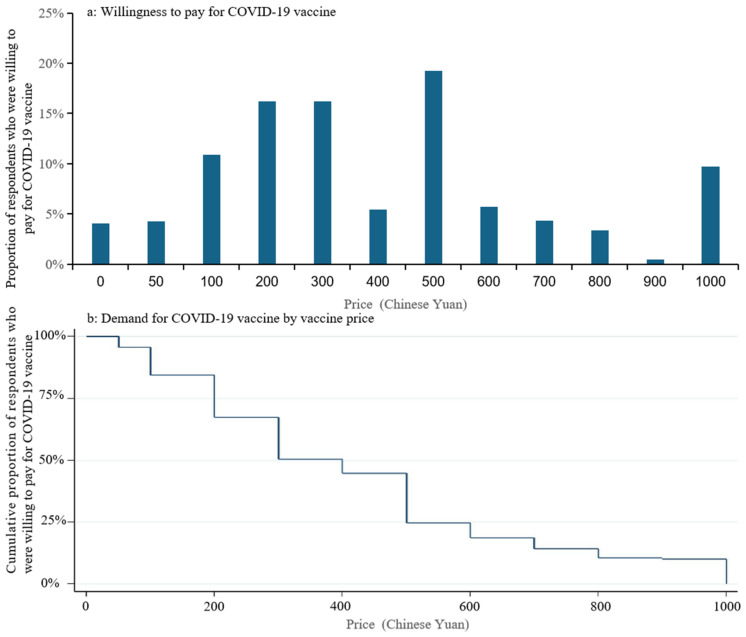
Willingness to pay and demand for COVID-19 vaccine: (**a**) Proportion of respondents who were willing to pay for COVID-19 vaccine at each price; (**b**) Cumulative proportion of respondents who were willing to pay for COVID-19 vaccine.

Overall, vaccine acceptance decreased as the cost of the COVID-19 vaccine increased. The proportion of respondents who were willing to be vaccinated against COVID-19 decreased from 92.50% if free to 83.90% if out-of-pocket payment required, and the proportion of those definitely willing to be vaccinated decreased even more from 69.0% to 47.90% (Figure 1a). Among all the respondents, there were 16.23%, 16.23% and 19.24% of respondents willing to pay maximum amounts of CNY 200 (USD 31), CNY 300 (USD 46) and CNY 500 (USD 77) for COVID-19 vaccine, respectively, and the median amount that respondents were willing to pay for a COVID-19 vaccine was CNY 300 (USD 46) (Figure 2a). Of respondents, 80.81% would get vaccinated if the price of the COVID-19 vaccine is set at CNY 200 (USD 31), and this proportion would decrease to 48.37% if the price of the COVID-19 vaccine is set at CNY 400 (USD 62) (Figure 2b).

**Figure 3:**
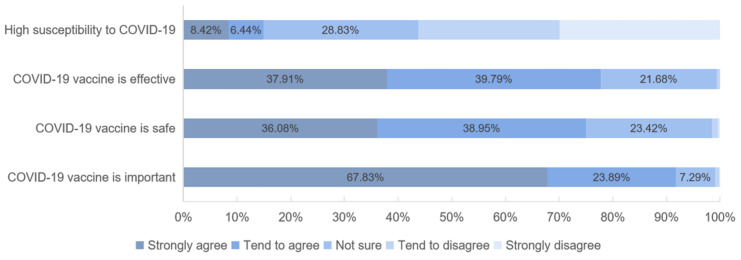
Confidence in COVD-19 vaccine and susceptibility to COVID-19 infection.

In addition, only 14.86% (*n* = 316) of the respondents considered themselves susceptible to COVID-19, but the majority (91.72%) perceived a COVID-19 vaccine as important (Figure 3). Of the respondents, 75.02% (*n* = 1595) and 77.70% (*n* = 1652) were confident in the vaccine’s safety and effectiveness, respectively.

### 3.3. Factors Associated with Acceptance and Willingness to Pay for COVID-19 Vaccine

Results from univariate analysis (Table 1) suggested that compared with their counterparts, the significantly higher proportion of respondents accepting COVID-19 vaccine were found among the younger, families with 3–4 members, those with higher education and more income, those living in Shanghai for less than 5 years and not working at a food market or supermarket, those frequent contact with local residents, and healthier migrants. After adjustment for covariates by multivariate logistic regression (Table 2), respondents not working at a food market or supermarket, those with more frequent contact with local residents (OR:1.79, 95% CI: 1.32–2.43), and those with good health (OR:1.78, 95% CI: 1.29–2.45) were significantly more likely to accept COVID-19 vaccination than their counterparts. In the additional model, the above associations still held true, and those who perceived a COVID-19 vaccination as important (OR:8.71, 95% CI:5.89–12.89), safe (OR:1.80, 95% CI:1.24–2.61), and effective (OR:2.66, 95% CI:1.83–3.87) were significantly more likely to accept COVID-19 vaccination.

Table 2 also displays the results of the ordered logistic regression assessing the factors associated with respondents’ WTP for the COVID-19 vaccine. In the basic model, female (OR:1.28, 95% CI: 1.09–1.51), the younger, those living in larger families, those reporting higher income, those with more frequent contact with local residents (OR:1.61, 95% CI: 1.37–1.89), and those with good health (OR:1.27, 95% CI: 1.05–2.55) had a significantly higher WTP for COVID-19 vaccine. In the additional model, respondents considered themselves susceptible to COVID-19 (OR:1.56, 95% CI:1.25–1.95), and those who perceived a COVID-19 vaccination as important (OR:1.88, 95% CI: 1.40–2.51) and effective (OR:1.91, 95% CI: 1.52–2.39) had a significantly higher WTP for the vaccine.

## 4. Discussion

Our study examined migrants’ attitudes toward, acceptance of and willingness to pay for a COVID-19 vaccine in Shanghai, China. We found a high acceptance of COVID-19 vaccination among this migrant population, with a majority (89.1%) willing to be vaccinated, no matter their socio-demographic characteristics. The main reasons for vaccine hesitancy included concerns about the vaccine’s safety and effectiveness, and a lack of awareness about the vaccine. Over 90.0% of migrants considered a COVID-19 vaccine as important, while only 75.0% and 77.7% agreed that the vaccine is safe and effective, respectively. In terms of WTP, migrants were willing to pay a median amount of CNY 300 (USD 46) for the COVID-19 vaccine.

We found the acceptance of the COVID-19 vaccine remained high in China, even with the low perceived susceptibility to COVID-19 in our surveys and the well-controlled local outbreaks [1]. Previous studies reported an acceptance rate of 90.0% to 91.3% for COVID-19 in China in March 2020 [15,21], which was higher than that in the US (57.6–69%), France (62.0%), the UK (80.0%) and Australia (85.5%) [15,16,18,20,33]. In China, the high acceptance of the COVID-19 vaccine was consistent with the high acceptance of other personal protective measurements, such as mask-wearing, hand-washing, and social distancing [34]. The COVID-19 vaccination is considered the most effective intervention to mitigate the pandemic; therefore, a high acceptance would be critical in improving the vaccine coverage. However, it remains unknown whether the high acceptance of the COVID-19 vaccine among migrants in Shanghai results in a high uptake of the vaccine once widely available. In the future, it is necessary to assess the real vaccination behaviors when the COVID-19 vaccine is available for the general population.

More interesting, the high acceptance of the COVID-19 vaccine was universal among Chinese migrants no matter their socio-demographic characteristics. This finding was different from that in other countries where socio-demographic factors including age, gender and education level were reported to be associated with respondents’ acceptance of the COVID-19 vaccine. In this study, however, no significant association was found between any socio-demographic factors and vaccine acceptance. The reason may lie in the effective communication between the government, media and the public, which contributed substantially to a high level of health awareness and promotion of preventive behavior [35]. In contrast, there was still a widespread debate on the effectiveness of preventive behaviors like mask-wearing worldwide despite it being validated that it can help reduce transmission [36]. Universal high vaccine acceptance in China highlighted the importance of effective health communication to promote public behaviors.

We found that respondents’ confidence in the importance, effectiveness and safety of COVID-19 vaccines was found as independent predictors of COVID-19 vaccine acceptance, highlighting the importance of maintaining vaccine confidence. Although most migrants recognized the importance of a vaccine against COVID-19, only three in four perceived the vaccine to be safe and effective, which are 10% lower than reported confidence in the safety (82.7%) and effectiveness (88.2%) of vaccines in general among the Chinese general population in 2019 [37]. Our estimate of confidence in the effectiveness of COVID-19 vaccines was lower than that (89.5%) in March 2020 [21]. Lack of confidence in the safety and effectiveness of COVID-19 vaccines were also listed as the top reasons for vaccine hesitancy among migrants in our survey. Reasons for low confidence in COVID-19 vaccines may be closely related to respondents’ doubts about the research and development of this vaccine. The short duration of clinical trials and the use of new bioscience technologies (e.g., mRNA vaccine) for the first time in human vaccines make the long-term safety and efficacy profiles of these newly developed vaccines unclear [38]. Any negative news related to vaccine failure is likely to have a detrimental impact on the public’s vaccine confidence [39]. In China, despite the COVID-19 vaccine being granted permission for emergency use, clinical trials were still ongoing at the time of this study, which may influence the public’s uncertainty about the vaccine. Therefore, public health information campaigns should be supported by the scientific community to address public concerns about the COVID-19 vaccine. Through creating a space for collaborative dialogue between the scientific community and the public, campaigns should not only aim to update the public with the latest information about COVID-19 vaccines, but to also build confidence in these vaccines and the vaccination program that delivers them.

In addition, cost also appears to play an important role in the acceptance of the COVID-19 vaccine. In this study, the proportion of respondents who were definitely willing to get a COVID-19 vaccine was 20% lower if they would pay for the vaccine themselves. Our survey documents a higher WTP (CNY 200–500, USD 31–77) for COVID-19 vaccines among migrants in Shanghai, China than in Malaysia (USD 31) [30], but lower than that in Chile (USD 184) [40]. Epidemic progression and differences in per capita income may explain the variation in WTP for the COVID-19 vaccine across regions [41]. The COVID-19 vaccine has been used in emergencies in China at a cost of CNY 400 (USD 61), which is higher than the amount most respondents were willing to pay in our survey. The current costs of COVID-19 vaccination could prove a barrier to vaccination for a large proportion (43%) of migrants. Consistent with results from other studies, income was also significantly associated with respondents’ WTP in this study [30,40]. The government should consider easing cost barriers, and providing free COVID-19 vaccination, especially to migrant residents with lower incomes. Fortunately, the Chinese government has promised free vaccination for the general population at the same time of approval for use on 30 December 2020.

Our study has several limitations. First, the cross-sectional study design limits inference on changes in respondent’s confidence and acceptance of COVID-19 vaccines over time. As the pandemic continues around the world, the public’s attitude towards the COVID-19 vaccine is also likely to change, and further studies are needed to investigate changes in COVID-19 vaccine acceptance after the vaccine is widely available to the public. Second, we did not assess vaccination behavioral outcomes, and social desirability may be present with self-reported data. Nonetheless, measures of acceptance are shown to more accurately predict health behavior than alternative variables [42]. Future research may help determine whether COVID-19 vaccine acceptance would lead to increased vaccination coverage in the real world. Third, there may be selection bias because we did not use random sampling. The results of our survey may not represent the opinions of the general migrant population.

## 5. Conclusions

Our study presents a universal high acceptance of COVID-19 vaccines among migrant populations in Shanghai, China, no matter their socio-demographic characteristics. Concerns about vaccine safety and effectiveness and the high costs of the COVID-19 vaccine may affect the acceptance and WTP, and thereby hinder the vaccination uptake in the future. To improve COVID-19 vaccine uptake, health communication is necessary to inform the public and alleviate their concerns about the safety and effectiveness of COVID-19 vaccines, and the cost barrier should be also eased.

## Figures and Tables

**Table 1:** Respondent characteristics and COVID-19 vaccine acceptance, *N* = 2126.

Characteristics	Total Sample, *N* (%)	COVID-19 Vaccine Acceptance		*p*-Value ^1^
		Accept, *N* (Row%)	Hesitant, *N* (Row%)	
Total	2126	1894 (89.09)	232 (10.91)	-
Gender				
Male	1070 (50.33)	957 (89.44)	113 (10.56)	0.601
Female	1056 (49.67)	937 (88.73)	119 (11.27)	
Age (years)				
≤25	491 (23.10)	433 (88.19)	58 (11.81)	<0.001
26–35	987 (46.43)	905 (91.69)	82 (8.31)	
36–45	375 (17.64)	337 (89.87)	38 (10.13)	
>45	273 (12.84)	219 (80.22)	54 (19.78)	
Marital status				
Single	620 (29.16)	549 (88.55)	71 (11.45)	0.406
Married	1441 (67.78)	1290 (89.52)	151 (10.48)	
Divorced or widow	65 (3.06)	55 (84.62)	10 (15.38)	
Number of family members				
1	292 (13.73)	254 (86.99)	38 (13.01)	0.022
2	455 (21.40)	404 (88.79)	51 (11.21)	
3	654 (30.76)	599 (91.59)	55 (8.41)	
4	355 (16.70)	321 (90.42)	34 (9.58)	
≥5	370 (17.40)	316 (85.41)	54 (14.59)	
Education				
Primary school or below	94 (4.42)	70 (74.47)	24 (25.53)	<0.001
Middle school	459 (21.54)	381 (83.19)	77 (16.81)	
High school	584 (27.47)	531 (90.92)	53 (9.08)	
Junior college	592 (27.85)	543 (91.72)	49 (8.28)	
Bachelor degree or above	398 (18.72)	369 (92.71)	29 (7.29)	
Monthly personal income (Chinese Yuan)				
≤2500	204 (9.60)	167 (81.86)	37 (18.14)	0.001
2501–5000	585 (27.52)	513 (87.69)	72 (12.31)	
5001–7500	726 (34.15)	654 (90.08)	72 (9.92)	
7501–10,000	347 (16.32)	314 (90.49)	33 (9.51)	
>10,000	264 (12.42)	246 (93.18)	18 (6.82)	
Years of local residence				
≤0.5	257 (12.09)	233 (90.66)	24 (9.34)	<0.001
0.5–1	283 (13.31)	259 (91.52)	24 (8.48)	
1–2	440 (20.70)	409 (92.95)	31 (7.05)	
2–5	481 (22.62)	440 (91.48)	41 (8.52)	
>5	665 (31.28)	553 (83.16)	112 (16.84)	
Workplace				
Food market or supermarket	334 (15.71)	256 (76.65)	78 (23.35)	<0.001
Small service industry such as catering or express delivery	514 (24.18)	464 (90.27)	50 (9.73)	
Manufacturing industry such as factory	266 (12.51)	238 (89.47)	28 (10.53)	
Company or government agency	768 (36.12)	724 (94.27)	44 (5.73)	
Unemployed	105 (4.94)	92 (87.62)	13 (12.38)	
Others	139 (6.54)	120 (86.33)	19 (13.67)	
Frequency of contact with local residents				
Frequent	1157 (54.42)	1053 (91.01)	104 (8.99)	0.002
Not frequent	969 (45.58)	841 (86.79)	128 (13.21)	
Self-rated health status				
Good	1705 (80.20)	1545 (90.62)	160 (9.38)	<0.001
Fair or poor	421 (19.80)	349 (82.90)	72 (17.10)	

^1^*p*-value from Chi-square test.

**Table 2:** Factors associated with acceptance and willingness to pay for COVID-19 vaccine.

Variables (Reference)	Logistic Regression for Vaccine Acceptance (Accept vs. Hesitant)		Ordered Logistic Regression for Willingness to Pay	
	Basic Model	Additional Model	BASIC Model	Additional Model
Female	1.07 (0.79–1.45)	1.05 (0.75–1.47)	1.28 (1.09–1.51) **	1.29 (1.10–1.51) **
Age (≤25, years)				
26–35	1.42 (0.91–2.23)	1.47 (0.90–2.40)	0.77 (0.61–0.97) *	0.73 (0.58–0.92) **
36–45	1.64 (0.92–2.92)	1.59 (0.85–3.00)	0.83 (0.62–1.12)	0.79 (0.59–1.06)
>45	1.30 (0.71–2.36)	1.56 (0.80–3.03)	0.60 (0.43–0.85) **	0.61 (0.43–0.86) **
Marital status (single)				
Married	1.45 (0.93–2.26)	1.18 (0.72–1.92)	0.99 (0.79–1.24)	0.98 (0.78–1.23)
Divorced or widow	0.85 (0.38–1.90)	1.08 (0.44–2.64)	1.40 (0.86–2.27)	1.44 (0.88–2.35)
Number of family members (1)				
2	1.37 (0.83–2.25)	1.28 (0.74–2.23)	1.16 (0.88–1.5)	1.03 (0.78–1.36)
3	1.41 (0.87–2.28)	1.12 (0.65–1.92)	1.30 (1.00–1.68) *	1.14 (0.88–1.48)
4	1.42 (0.83–2.43)	1.28 (0.71–2.33)	1.33 (1.00–1.77)	1.17 (0.87–1.56)
≥5	0.97 (0.59–1.59)	0.95 (0.55–1.66)	1.55 (1.16–2.06) **	1.42 (1.06–1.89)*
Education (primary school or below)				
Middle school	1.11 (0.62–1.97)	1.04 (0.54–2.02)	0.92 (0.59–1.44)	0.93 (0.60–1.46)
High school	1.69 (0.90–3.18)	1.57 (0.77–3.20)	0.97 (0.61–1.52)	0.94 (0.60–1.48)
Junior college	1.33 (0.67–2.62)	1.26 (0.58–2.71)	0.91 (0.57–1.46)	0.88 (0.55–1.41)
Bachelor degree or above	1.40 (0.66–2.94)	1.64 (0.71–3.80)	0.70 (0.43–1.14)	0.70 (0.43–1.15)
Monthly personal income (≤2500 Chinese Yuan)				
2501–5000	1.38 (0.86–2.20)	1.37 (0.80–2.32)	1.29 (0.96–1.74)	1.25 (0.93–1.68)
5001–7500	1.32 (0.81–2.15)	1.23 (0.71–2.14)	2.12 (1.56–2.88) **	2.03 (1.49–2.76) **
7501–10,000	1.39 (0.79–2.46)	1.01 (0.54–1.90)	3.68 (2.62–5.17) **	3.34 (2.37–4.70) **
>10,000	1.87 (0.96–3.64)	1.64 (0.78–3.44)	4.07 (2.82–5.89) **	3.96 (2.73–5.74) **
Years of local residence (≤0.5 years)				
0.5–1	0.84 (0.45–1.56)	0.79 (0.40–1.57)	1.31 (0.97–1.77)	1.27 (0.94–1.72)
1–2	0.94 (0.53–1.69)	0.91 (0.48–1.73)	1.33 (1.01–1.76) *	1.31 (1.00–1.74)
2–5	0.58 (0.33–1.04)	0.59 (0.31–1.11)	1.08 (0.81–1.44)	1.03 (0.77–1.37)
>5	0.37 (0.22–0.64) **	0.43 (0.24–0.80) **	0.94 (0.71–1.24)	0.98 (0.74–1.29)
Workplace (food market or supermarket)				
Small service industry	2.41 (1.53–3.78) **	2.18 (1.32–3.62) **	1.10 (0.84–1.45)	0.96 (0.73–1.27)
Manufacturing industry	2.20 (1.32–3.67) **	1.99 (1.11–3.58) *	0.75 (0.55–1.02)	0.68 (0.50–0.93) *
Company or government agency	4.03 (2.49–6.52) **	3.13 (1.83–5.35) **	0.85 (0.65–1.11)	0.78 (0.60–1.03)
Unemployed	2.18 (1.07–4.44) *	1.64 (0.76–3.55)	0.74 (0.48–1.13)	0.67 (0.43–1.02)
Others	1.65 (0.90–3.03)	1.37 (0.69–2.70)	0.97 (0.67–1.41)	0.94 (0.65–1.37)
Frequent contact with local residents	1.79 (1.32–2.43) **	1.48 (1.05–2.09) *	1.61 (1.37–1.89) **	1.48 (1.26–1.75) **
Good self-rated health	1.78 (1.29–2.45) **	1.40 (0.98–2.00)	1.27 (1.05–1.55) *	1.12 (0.92–1.36)
High susceptibility of COVID-19		1.59 (0.91–2.80)		1.56 (1.25–1.95) **
Confident in importance of COVID-19 vaccine		8.71 (5.89–12.89) **		1.88 (1.40–2.51) **
Confident in safety of COVID-19 vaccine		1.80 (1.24–2.61) **		1.06 (0.86–1.32)
Confident in effectiveness of COVID-19 vaccine		2.66 (1.83–3.87) **		1.91 (1.52–2.39) **

Notes: Odds ratio and 95% confidence intervals were presented. Significance level: ** *p* < 0.01, * *p* < 0.05.

## Data Availability

The corresponding author had full access to all the data in the study and had final responsibility for the decision to submit for publication.
